# Comparison of Ultrasonography and Scintigraphy as Localization Techniques in the Preoperative Evaluation of Primary Hyperparathyroidism

**DOI:** 10.7759/cureus.82021

**Published:** 2025-04-10

**Authors:** Nahomi S Siordia Cruz, Carlos F Gallegos De Luna, Isac I Ramírez-Preciado, Jacob J Zavala Mejía, Gloria A Peña Montañez, Manuel Sánchez González, Gonzalo Delgado Hernández, José V Pérez Navarro

**Affiliations:** 1 General Surgery, Centro Médico Nacional de Occidente, Instituto Mexicano del Seguro Social (IMSS), Guadalajara, MEX

**Keywords:** hyperparathyroidism primary, localization techniques in primary hyperparathyroidism, parathyroid neoplasms, parathyroid scintigraphy, parathyroid ultrasonography

## Abstract

Primary hyperparathyroidism (pHPT) is the most common endocrine disorder responsible for hypercalcemia in non-hospitalized patients. When indicated for parathyroidectomy for pHPT, preoperative imaging is recommended to localize the affected parathyroid glands, including neck ultrasonography and 99m-Tc-sestaMIBI scintigraphy. The aim of this study was to investigate and compare the findings obtained by ultrasonography and scintigraphy in the preoperative evaluation. A retrospective review was conducted on all patients who underwent partial parathyroidectomy for a diagnosis of pHPT between January 2022 and December 2024 in a specialized center in Mexico. The locations according to scintigraphy and ultrasound were compared using the McNemar test, and specificity, sensitivity, negative predictive value (NPV), and positive predictive value (PPV) were determined. A significant difference was observed only in the case of non-localization of affected glands (p<0.0001).

For scintigraphy, sensitivity was 100%, specificity 60.70%, PPV 73.7%, and NPV 100%. For ultrasonography, sensitivity was 60%, specificity 87.5%, PPV 93.2%, and NPV 25.93%. The concordance observed between the preoperative localization findings obtained by 99m-Tc-sestaMIBI scintigraphy and ultrasonography in patients with pHPT highlights the enduring value of ultrasonography as a preoperative tool, due to its accessibility and cost-effectiveness.

## Introduction

Primary hyperparathyroidism (pHPT) is the most common endocrine disorder responsible for hypercalcemia in non-hospitalized patients. The incidence is approximately 65.5 and 24.7 per 100,000 person-years in women and men, respectively [1-3]. The diagnosis of pHPT is based on biochemical parameters, reserving imaging tests for patients considered for surgery [4,5]. In the case of hypercalcemic pHPT, an elevated serum calcium level is required, with an elevated or inappropriately normal parathyroid hormone (PTH) level on two occasions, with at least a two-week interval between measurements [4]. Furthermore, it is recommended that the initial biochemical evaluation include serum levels of creatinine, phosphorus, and 25-hydroxyvitamin D [5].

In patients indicated for parathyroidectomy, preoperative imaging is recommended to localize the affected parathyroid glands [5]. Preoperative imaging modalities include neck ultrasonography, 99m-Tc-sestaMIBI scintigraphy, and more recently, four-dimensional computed tomography with contrast [6]. With adequate preoperative imaging, selective parathyroidectomy, either combined or not with intraoperative PTH monitoring, achieves high success rates when performed by experienced surgeons [4,7].

Cervical ultrasonography, performed by a specialist experienced in parathyroid imaging, is the most cost-effective and accessible imaging modality. Therefore, it is recommended as a method for locating parathyroid disease and assessing potential concomitant thyroid pathology [5,8]. However, the sensitivity of ultrasonography for localization is typically reported as low but variable, ranging from 19% to 84%, with 85% specificity [9].

Scintigraphy utilizes methoxy-isobutylisonitrile (sestaMIBI) labeled with 99m-technetium, which is absorbed by mitochondria-rich cells, allowing for the evaluation of deep and ectopic cervical glands, including those located in the mediastinum. Additionally, it offers relatively low exposure to ionizing radiation and the ability to assess the function of autotransplanted tissue in the forearm or other areas [5,10]. Its utility is limited by the inability to examine the thyroid, the risk of false positives associated with thyroid nodules, and its low effectiveness in detecting multiglandular disease [10].

The aim of this study was to investigate and compare the findings obtained by ultrasonography and 99m-Tc-sestaMIBI scintigraphy in the preoperative evaluation of primary hyperparathyroidism.

## Materials and methods

A retrospective review was conducted on all patients who underwent partial parathyroidectomy for a diagnosis of pHPT between January 2022 and December 2024 at the specialized Endocrine Surgery Department of the Centro Médico Nacional de Occidente, part of the Instituto Mexicano del Seguro Social (IMSS), in Guadalajara, Mexico. Inclusion and exclusion criteria are outlined in Table 1. Initially, 69 patients were reviewed, with 11 excluded for failing to meet the inclusion and exclusion criteria, resulting in a final sample of 58 patients. The diagnosis, treatment indication, and management of pHPT were based on the criteria outlined in the Fifth International Workshop on the Evaluation and Management of Primary Hyperparathyroidism [4] and the American Association of Endocrine Surgeons Guidelines for the Definitive Management of Primary Hyperparathyroidism [5]. 

**Table 1:** Inclusion and exclusion criteria for enrolling patients in the study

Inclusion criteria	Exclusion criteria
Patients aged 18 years or older	Patients who had undergone prior parathyroid surgery
Both genders	Patients who did not achieve cure
Diagnosis of primary hyperparathyroidism	Patients lacking the required imaging studies
Underwent surgical treatment	
Availability of preoperative neck ultrasound and scintigraphy	

After the diagnosis of pHPT was established and surgical treatment indicated, preoperative imaging was performed using ultrasound and scintigraphy for the localization of the affected parathyroid glands. Ultrasound was performed using a 4-8 MHz linear transducer (Philips). The patients were placed in a supine position, with the neck extended and shoulders relaxed, and both longitudinal and transverse images were captured. For the scintigraphy imaging, a dual-phase technique was used, where 20 mCi (740 MBq) of 99m-Tc-sestaMIBI was administered. The 99m-Tc window was set to 140 keV at 20%, and images were obtained at 20 and 120 minutes after the administration of the radiotracer. All ultrasound images were reviewed and interpreted by the institution's imaging department, and the scintigraphy was interpreted by the nuclear medicine department.

The surgical procedure was performed by surgeons specialized in parathyroid surgery, registered in the endocrine surgery department, with histopathological study of the affected parathyroid glands to confirm the presence of parathyroid tissue.

Four possible locations for the affected gland were established: a) Right superior, b) Left superior, c) Right inferior, and d) Left inferior. Additionally, two other variables were determined: the presence of multiglandular disease and the non-detection of the affected glands. The correct localization of the affected parathyroid glands was determined based on surgical findings and histopathological confirmation. Data was collected on the patients' age, gender, preoperative PTH, vitamin D, and serum calcium levels, along with the variables mentioned. The locations according to scintigraphy and ultrasound were compared using the Bhapkar χ² test, symmetry of differences was evaluated using the Bowker test, and each location was analyzed separately with the McNemar test, applying the Bonferroni adjustment. Concordance analysis between uniglandular or multiglandular disease between surgical findings and ultrasound and scintigraphy findings determined specificity, sensitivity, negative predictive value (NPV), and positive predictive value (PPV). A 95% confidence interval was established. The platform used for the analysis was Microsoft® Excel® (version 2412, build 16.0.18324.20092; Microsoft Corporation, Redmond, USA) 64-bit, and IBM SPSS Statistics for Windows, Version 22 (Released 2013; IBM Corp., Armonk, New York, United States).

## Results

**Figure 1:**
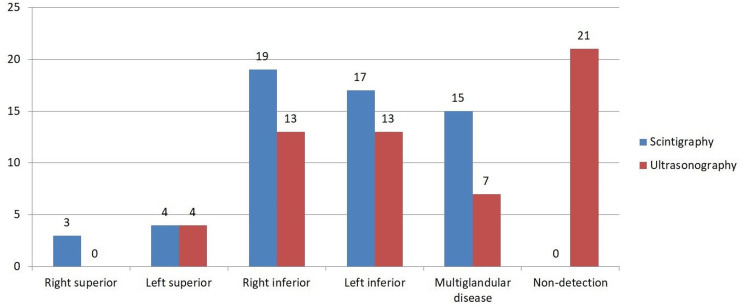
Frequency for each category according to scintigraphy and ultrasonography

The analysis included 58 patients, 10 (18.9%) men and 48 (84.4%) women. The characteristics of the study population are outlined in Table 2. Initially, the findings obtained by ultrasonography and scintigraphy were analyzed, categorizing them into the six established categories, which included the four possible locations of affected glands in cases of uniglandular disease, the presence of multiglandular disease, and the absence of relevant findings. The frequency distribution is illustrated in Figure 1.

**Table 2:** General characteristics of the population

Characteristic (n=58)	Values
Sex	
Male	10 (18.9%)
Female	48 (84.4%)
Age (years)	57.9
Preoperative laboratory studies	
PTH (pg/ml)	397.2
Serum calcium (mg/dl)	12.2
25-hydroxyvitamin D (ng/ml)	27.1
Diagnostic	
Adenoma	13 (22.40%)
Hyperplasia	45 (77.60%)

The Bhapkar χ² test was conducted as a general marginal homogeneity test, resulting in a χ² value of 43.474 with a significance of p = 0.00003. The Bowker symmetry test yielded a χ² of 29.0, with a p-value of 0.0161, indicating asymmetrical heterogeneity. Subsequently, the McNemar test was performed for each localization category, obtaining χ² values and significance adjusted for Bonferroni. The results are presented in Table 3. A significant difference was observed only in the case of non-localization of affected glands (p<0.0001).

**Table 3:** McNemar test with Bonferroni adjustment for each category according to scintigraphy and ultrasonography * p < Bonferroni-adjusted significance criterion of 0.010.

Categories	Frequency		Chi-squared	P*
	Scintigraphy	Ultrasonography		
Right superior	3 (5.2%)	0	3	1
Left superior	4 (6.9%)	4 (6.9%)	0	1
Right inferior	19 (32.8%)	13 (22.4%)	2.571	1
Left inferior	17 (29.2%)	13 (22.4%)	1.600	1
Multiglandular disease	15 (25.9%)	7 (12.1%)	5.333	4.939
Non-detection	0	21 (36.2%)	21.0	2

The presence of uniglandular or multiglandular disease was analyzed by comparing the findings between scintigraphy and ultrasonography with the surgical results, as shown in Table 4. For scintigraphy, sensitivity was 100% (95% CI, 88.65% - 100%), specificity was 60.70% (95% CI, 42.41% - 76.43%), PPV was 73.7%, and NPV was 100%. For ultrasonography, sensitivity was 60% (95% CI, 46.18% - 72.39%), specificity was 87.5% (95% CI, 52.91% - 97.76%), PPV was 93.2% (95% CI, 83.81% - 99.43%), and NPV was 25.93% (95% CI, 13.17% - 44.68%).

**Table 4:** Accuracy parameters obtained in ultrasonography and scintigraphy as preoperative localization techniques

Accuracy Parameters	Scintigraphy	Ultrasonography
Sensitivity	100%	60%
Specificity	60.70%	87.50%
Positive predictive value	73.70%	93.20%
Negative predictive value	100%	25.93%

## Discussion

The parathyroid glands are typically located at the four poles of the thyroid gland; however, they can also be found in other locations, making their transsurgical identification challenging. Therefore, the use of imaging studies is recommended for evaluation and preoperative planning [11], with ultrasonography and 99m-Tc-sestaMIBI scintigraphy being the most commonly used modalities [12,13].

Since there is no current consensus regarding the ideal imaging modality for locating affected parathyroid glands [5], as well as the reported differences in efficacy between modalities, and considering that the choice is often based on availability, surgeon experience, and preference [14], this study aimed to compare the two modalities most readily available at our specialized center.

The characteristics of the studied population are consistent with the described epidemiology, showing a predominant female-to-male ratio of 3 to 4 times [3], with a peak prevalence age between 50 and 60 years [15]. The most common histological etiology was parathyroid adenoma [15,16], and typical biochemical characteristics were found as follows: elevated PTH, hypercalcemia, and normal vitamin D levels [4,17].

The results from the Bhapkar test, used to evaluate the homogeneity of findings in the localization of affected glands, demonstrated a significant difference (p = 0.00003) between ultrasonography and scintigraphy. However, the Bowker symmetry test was performed to assess whether this discrepancy was consistent across all localization categories, revealing asymmetry between the categories (p = 0.0161). Detailed analysis using the Bonferroni-adjusted McNemar test identified a significant difference only when ultrasonography failed to locate the affected parathyroid gland (p = 0.00021).

When evaluating the sensitivity and specificity values of ultrasonography, the results were similar to those previously described in the literature (Table 3), with a low sensitivity of 60%, which falls within the reported range of 55.3% to 77% [6,18,19], providing a reliable sensitivity value for our population and setting, since the variability between centers and populations is high. The specificity was 87.5%, with a PPV of 93.2%, which is close to the sensitivity of 85.2% and the PPV of 95.7% found in previous meta-analyses [6,19]. In contrast, scintigraphy demonstrated 100% sensitivity, but its specificity decreased, even falling below that of ultrasonography at 60.7%, which is lower than previous reports that place the value between 82% and 86.8% [20,21]. Similarly, its PPV was lower than reported, at 73.7% versus 95% [20].

Ultrasonography remains effective for locating parathyroid adenomas close to the thyroid gland or in the superior cervical portion of the thymus, but its effectiveness significantly decreases when trying to identify adenomas located behind the trachea or esophagus, as well as ectopic glands [22]. It is important to note that both techniques have been found to be inadequate for patients with a history of cervical surgery, concomitant thyroid disease, or unfavorable anatomical conditions [23].

Currently, techniques such as four-dimensional tomography or SPECT-CT have demonstrated better sensitivity and specificity [6,24], and even performance can be improved by combining scintigraphy and ultrasonography. However, their availability remains limited, and costs are high. Therefore, it is crucial to continue investigating the application of more accessible and cost-effective methods [8,24,25].

Our main limitation is the availability of studies that currently promise a higher sensitivity and specificity value, such as 4D CT or SPECT CT. Likewise, although we are a regional referral center for the management of pHPT, our cohort is small compared to larger and multicentric studies.

## Conclusions

The concordance observed between the preoperative localization findings obtained by 99m-Tc-sestaMIBI scintigraphy and ultrasonography in patients with pHPT, along with the correlation with surgical findings, highlights the enduring value of ultrasonography as a preoperative tool, due to its accessibility and cost-effectiveness.
